# “More Than Buying Extra Fruits and Veggies, Please Hide the Fats and Sugars”: Children’s Diet Latent Profiles and Family-Related Factors

**DOI:** 10.3390/nu13072403

**Published:** 2021-07-13

**Authors:** Beatriz Pereira, Cátia Silva, José Carlos Núñez, Pedro Rosário, Paula Magalhães

**Affiliations:** 1 Department of Applied Psychology, University of Minho, 4710-052 Braga, Portugal; beatriznpereira94@gmail.com (B.P.); catiasbsilva@gmail.com (C.S.); prosario@psi.uminho.pt (P.R.); 2 Department of Psychology, University of Oviedo, 33003 Oviedo, Spain; jcarlosn@uniovi.es

**Keywords:** diet profiles, income, food availability, parental encouragement, elementary school-aged children

## Abstract

Promoting children’s healthy diets is a key public health priority. Family can play a relevant role in children’s eating patterns. The goals of the current research were to identify different latent diet profiles in children based on their food consumption and to assess the relationship between profiles and family-related factors. A total of 678 school-aged children from the fifth and sixth grades participated. The study design was cross-sectional and questionnaire based. Research assessed healthy (fruit and vegetables) and unhealthy (fast food, sugar-sweetened beverages, and candies) food consumption and family-related factors. A latent profile analysis and multivariate data analysis were developed. Four diet profiles were identified: Combined Diet, Mainly Healthy Diet, Mainly Unhealthy Diet, and Very Unhealthy Diet. Nearly half of the children (45.22%) showed a Combined Diet profile, meaning that they reported eating nearly the same amount of healthy and unhealthy types of foods. Associations between the diet profiles, family income, and food availability were found. For example, the Mainly Healthy Diet profile was statistically associated with a higher family income and less access to unhealthy foods. The present study reinforces the idea that profiling diets can allow for a tailored healthy eating intervention model according to the specific needs of each diet profile.

## 1. Introduction

The promotion of a healthy diet is a key public health priority for the prevention of chronic diseases (e.g., obesity, diabetes, heart disease, and cancer) and the maintenance of health and well-being from early childhood into late adulthood [1]. Promoting a healthy diet early in life is vital because eating habits developed during childhood are likely to continue throughout adulthood [2]. Following this line of reasoning, the World Health Organization establishes eating guidelines for children, with great focus on the intake of fruits and vegetables (F/V) and on the restriction of fat and sugar consumption [3]. These guidelines are also followed by the Portuguese Directorate-General for Health [4]. However, evidence that children are not meeting the recommendations (e.g., five pieces of F/V per day) is extensive, including in Portugal [5,6,7]. In fact, children are still consuming large amounts of energy-dense foods, fat, sugar, and salt, and consuming low amounts of fiber, vegetables, fruits, and whole grains [3].

Family, as the primary socializing context for children, plays a crucial role in the promotion of a healthy diet [8]. More specifically, a family’s characteristics are likely to influence children’s diet-related opportunities and resources [9]. For example, children from low-income families are less likely to exhibit healthy dietary behaviors compared to children from high-income families [10,11,12]. The constrained financial resources and opportunities to buy healthy foods may contribute to children falling short of healthy dietary patterns [13,14]. Within Portuguese families, this scenario is no exception. Over the years, food intake and availability has changed considerably in Portugal. An increased access to fat and sugar products is contributing to changes in dietary patterns [15,16]. In fact, families are able to provide their children with dietary energy at a reduced cost by relying on fats and sweets, as fruits and vegetables imply an increase of costs [14,17]. Consequently, children from low-income families may have less access to healthy foods at home. Having a limited variety of foods at home may narrow children’s dietary choices, with the likelihood of selecting a healthy or unhealthy snack being low when the availability of these types of foods is low [18,19,20]. Importantly, the recent literature has found that the availability of healthy food at home is strongly correlated to parental encouragement of healthy eating [21]. The latter variable is expected to influence children’s diets; however, research on this topic is still limited [8,9,22]. Grounded by this literature review, it seems important to consider family-related factors within the study of children’s diets, in efforts to further understand the roles that they may play. Hopefully, these data will help to organize interventions tailored to families and children’s healthy eating needs.

Recently, it has been proposed that the study of eating behaviors should not only focus on describing diets as healthy or unhealthy, but also on identifying eating configurations using the concept of profiling [23,24]. Accordingly, several methods have been used with different target populations [25,26] to search for diet profiles (i.e., analyze distinct dietary patterns). Regarding children, although patterns of health-related behaviors (e.g., sleep, screen time, diet) have been studied, the spectrum of diet profiles that they may present has been receiving less attention from researchers, i.e., the spectrum ranging from very healthy to very unhealthy diets [23,24,27]. This proposition underlines the need for further research in this topic. We believe profiling children’s diets and examining how the different profiles are associated with key family variables will allow for the development of tailored approaches to improve children’s diet quality.

The present study aims to identify and describe children’s diet profiles using latent class analysis and to further analyze the relationship of family-related factors (e.g., income, food availability at home) with latent class membership. Rooted in the available literature on the topic, the following hypotheses were set:

**Hypothesis** **H1.** *We expect to identify three distinct diet profiles according to children’s reported food consumption: mainly healthy; mainly unhealthy; and balanced, i.e., a combined similar amount of healthy and unhealthy foods.*

**Hypothesis** **H2.** *We expect to find a relationship between family income and diet profiles. The greater the family income, the more likely children are to have a mainly healthy profile, and the smaller the family income, the more likely children are to have a mainly unhealthy profile.*

**Hypothesis** **H3.** *We expect to find a relationship between foods available at home and diet profiles. The more access to healthy food, the more likely children are to have a mainly healthy profile; the more access to unhealthy food, the more likely children are to have a mainly unhealthy profile; and, finally, the more access to both types of food, the more likely children are to have a balanced diet profile.*

**Hypothesis** **H4.** *We expect to find a relationship between parental encouragement of healthy eating and diet profiles. The higher the parental encouragement, the more likely children are to have a mainly healthy profile, and the lower the parental encouragement, the more likely children are to have a mainly unhealthy profile.*

## 2. Materials and Methods

### 2.1. Participants and Procedure

The participants were selected from five public schools in both rural and urban areas of Portugal. The data of the present study are part of a large research project (CEICSH 032/2019) with students from the fifth and sixth grades. The students from these two grades of five schools were invited, and, eventually, 744 students accepted the offer to participate in the research (85% acceptance rate). Late elementary school students were chosen as participants, as the formation of habits and behaviors takes place during childhood [28]. Moreover, previous research has suggested that this age is the ideal stage to discuss, learn, and enact behavior changes, including in the healthy eating domain [29,30]. From the initial pool of participants, 66 (8.87%) children had to be excluded due to having special educational needs. These students were excluded from the data analyses because we were only interested in accessing the relationships between diet profiles and a set of family-related variables (e.g., parental encouragement) irrespective of learning disabilities or of the capacity to express themselves. Finally, 678 Portuguese students participated in this data collection session. Written informed consent was asked for from children and parents/caregivers before data collection took place. To guarantee the confidentiality and anonymity of the data, researchers assigned individual codes to each participant. Data collection took place during regular classes, and children took approximately 20 min to complete the questionnaires. From this pool, 14 (2.06%) participants did not complete all the instruments. Thus, the final sample comprised 664 students with a mean age of 10.92 and a SD = 0.83 (response rate of 97.94%). Of these participants, 311 (49.85%) were female. Regarding family income (M = 2.92, SD = 1.24), 21.3% of the participants were from families with extremely very low incomes, 18.7% were from families with very low incomes, 7.2% were from families with low incomes, and 52.8% were from families with medium to high incomes (see Measures section for more details about family income calculation).

### 2.2. Measures

#### 2.2.1. Socio-Demographic Questionnaire

Participants were asked about their sex, age, and grade.

#### 2.2.2. Previous Day Food Intake Questionnaire (PDFIQ)

This instrument consists of food illustrations for assessing the foods that children ate at different meals throughout the previous day (i.e., breakfast, mid-morning snack, lunch, afternoon snack, dinner, and supper) [31]. The instrument consists of six images, one for each meal, illustrated with 21 individual foods and some food groups, specifically, dry beans, rice, milk, coffee with milk, chocolate milk, cheese, yogurt, beef or poultry, pasta, bread or crackers, French fries, pizza or hamburger, leafy vegetables, starchy vegetables, vegetable soup, fruits, sweets, chips, fish/seafood, soft drinks, and fruit juices [31]. For each image of a meal, children were asked to circle the foods that they ate throughout the previous day for the corresponding meal. Considering the goals of the present study, responses for each image were scored based on the frequency in which healthy foods were consumed throughout the day (i.e., the sum of the frequencies in the six illustrations) and the frequency in which unhealthy foods were consumed throughout the day (i.e., the sum of the frequencies in the six illustrations). The healthy foods category comprises the leafy vegetables, vegetable soup, fruits, and fruit juices from the PDFIQ illustrations. The unhealthy foods category comprises the chocolate milk, French fries, pizza or hamburger, sweets, chips, and soft drinks from the PDFIQ illustrations. For the present sample, the average consumption of healthy foods was 2.54 per day (SD = 1.72), and the average consumption of unhealthy foods was 1.46 per day (SD = 1.50). Additionally, only 16.2% of the children reported having consumed five or more F/V per day in their diet. The reliability of the scale is moderate for healthy (KR-20 = 0.673) and unhealthy (KR-20 = 0.619) food intake.

#### 2.2.3. Family Income

Income was chosen as a measure of financial resources due to research suggesting using it as a simple indicator [32] and its influence on the individual’s capability to purchase food [13]. The families’ incomes were assessed through students’ School Social Action Levels. This comprises three levels of family income, corresponding to the family’s annual income up to a certain amount: level A, up to EUR 3050.32 per year; level B, up to EUR 6100.64 per year; and level C, up to EUR 9150.96 per year. Families with an income greater than EUR 9150.96 per year do not receive School Social Action, as they are classified as high income [33]. Please note that in Portugal the minimum wage in 2019 was EUR 600 per month, totaling a gross annual income of EUR 8400 [34]. Thus, for the current study, family income was assessed as 1 = extremely low income, 2 = very low income, 3 = low income, 4 = medium to high income.

#### 2.2.4. Food Availability at Home

To assess food availability at home, a checklist comprised of eight food groups was used. The checklist consisted of eight images (i.e., cereals and derivatives; vegetables; fruits; legumes; dairy products; meat, fish, and eggs; candies and sugar-sweetened beverages (sugars); chips and fast-food (fats)), and children were asked to circle the groups of foods that they had available at home. Considering the present study goals, food availability was assessed as follows: healthy food availability (0 = no access to either fruit or vegetables; 1 = access to fruits or to vegetables; 2 = access to both F/V), unhealthy food availability (0 = no access to either fats or sugars; 1 = access to fats or to sugars; 2 = access to both fats and sugars).

#### 2.2.5. Parental Encouragement of Healthy Eating

To evaluate the parental encouragement of healthy eating, an adapted version of the “Attitudes and Perceptions about Health” Instrument [35] was used. The present instrument consisted of six statements about children’s perceptions regarding parental encouragement of healthy eating (e.g., My parents say that I will have more energy if I eat fruits and vegetables). Each item was scored as true or false, and the answers marked to be true were summed to create a composite score that ranged from 0 to 6, with higher scores implying more parental encouragement towards healthy eating. The reliability of the scale is 0.77.

### 2.3. Data Analysis

Data were analyzed in several steps. First, the statistical properties of the variables were computed in SPSS 27. Second, using iterative maximum likelihood estimation techniques with robust standard error (MLR), a latent profile analysis was performed to identify the number of unobserved subgroups comprising children of similar diet behaviors [36]. In mixture modeling, indicator variables were used to identify an underlying latent categorical variable. To identify diet profiles, two measures of food consumption (i.e., healthy and unhealthy food) were used on Mplus 7.11 [37]. Both measures were standardized before data analysis. Considering the potential relationship between sex and food consumption, the variable sex was included as a covariate in the estimation of latent classes. To determine the model that best described the relationship between variables of food consumption, the estimation was carried out by adding successive latent classes to the model [38]. Typically, the model that best fits data is the one using the optimal number of classes to describe the existing relations between variables [39]. For the selection of the best model, the model fitting assessment and model comparisons utilized the adjusted Lo–Mendell–Rubin test (LMRT), Akaike’s Information Criterion (AIC), Bayesian Information Criterion (BIC), sample-size adjusted BIC (SSA-BIC), the entropy value, and class size as measures of fit when comparing different numbers of latent class models. Significant *p*-values associated with LMRT indicate significant improvement in model fit relative to the solution, with one less class. Lower values of AIC, BIC, and SSA-BIC indicate a better fit of the model. To determine the classification accuracy of the selected model, the a posteriori probabilities and the entropy statistic were calculated. This statistic takes values between zero and one. Note that the closer to one, the more accurate the classification (values higher than 0.80 indicate good classification quality) [40]. Lastly, using the new classification classes (i.e., diet profiles) (obtained with the sentence SAVE = CPROBABILITIES in Mplus syntax) multivariate data analyses were conducted to predict the outcome variables (i.e., income, food availability, and parental encouragement of healthy eating). The criteria by Cohen were used to assess the size of the mean differences (small: *d* = 0.20; medium: *d* = 0.50; large: *d* = 0.80) [41].

## 3. Results

### 3.1. Descriptive Statistics

Table 1 shows the descriptive statistics and Pearson correlations between variables. Most correlations are statistically significant (58%). Unexpectedly, healthy food consumption is positively associated with unhealthy food consumption. This indicates that the more children eat healthy food, the more they also eat unhealthy food. Children with unhealthy eating patterns tend to be from low-income families and tend to have more access to unhealthy foods. Conversely, children with healthy eating patterns tend to have parental encouragement of healthy eating and tend to have less access to unhealthy foods. Lastly, variables are normally distributed [42].

### 3.2. Latent Profile Analysis

#### 3.2.1. Selection of the Best Model

Several latent profile models were fit to the data (models from two to five classes). The variables used to create the classes were healthy diet (e.g., fruit) and unhealthy diet (e.g., fast food). When fitting the models, we assumed that the variances could differ between the indicators within each group and set a restriction that the variances should be the same between groups. Likewise, the independence between indicators, both within and between groups, was imposed as a restriction. As aforementioned, considering the differences in eating habits between boys and girls, the variable of sex was included as a covariate when adjusting the models (the effect of gender on the establishment of categories and distribution of individuals between them). Results are depicted in Table 2.

There was no statistical justification to go beyond a four latent class model based on the best fit measures (distortions in the parameter estimation and entropy). Therefore, Table 2 shows information for the model fit up to four classes. This model was selected as the best fit for several reasons, as follows. First, fit statistics IC, BIC, SSA-BIC, and the LMRT-Test indicated that the model with four classes is better than that with three classes. Second, despite the entropy of the four-class model being somewhat smaller than that of the three-class model, the difference is small. Nevertheless, the entropy of the four-class model is still very good. Third, theoretically, the four-class model is more adequate than the three-class model since, in the latter model, the group of children with a very unhealthy diet profile does not emerge. We believe that, despite there being a small percentage of children in this profile, it is still relevant to consider. In sum, the four-class model was selected because: (i) it shows a statistically better fit, (ii) it is theoretically valid, and (iii) it is a high-quality model when it comes to classifying children within classes. The probability of each subject being classified within the class to which they were assigned is excellent overall (0.858) and for each individual class (class 1 = 0.835; class 2 = 0.992; class 3 = 0.980; class 4 = 0.974). Lastly, as aforementioned, models were fit with sex as a covariate (sex regressed on class). Data indicate that sex did not affect the results of the fit of the models. In other words, the allocation of participants to the classes, or the entropy, is not related to the inclusion of sex as a covariate.

#### 3.2.2. Description of the selected latent class model

Table 3 shows the relevant information on the four-class model.

**Figure 1:**
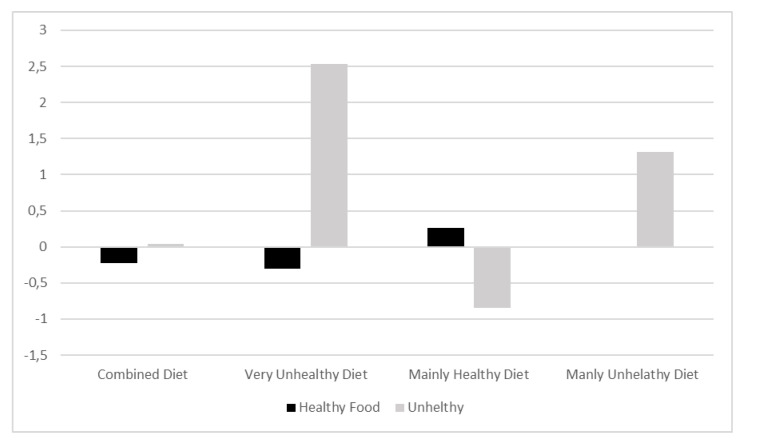
Graphical representation of diet profiles: Combined diet (*n* = 303; 45.22%), Very unhealthy diet (*n* = 36; 5.37%), Mainly healthy diet (*n* = 232; 34.62%), Mainly unhealthy diet (*n* = 99; 14.77%).

Figure 1 depicts the levels of healthy and unhealthy food consumption corresponding to the four classes or diet profiles. Values of both variables were standardized (M = 0; SD = 1).

**Figure 2:**
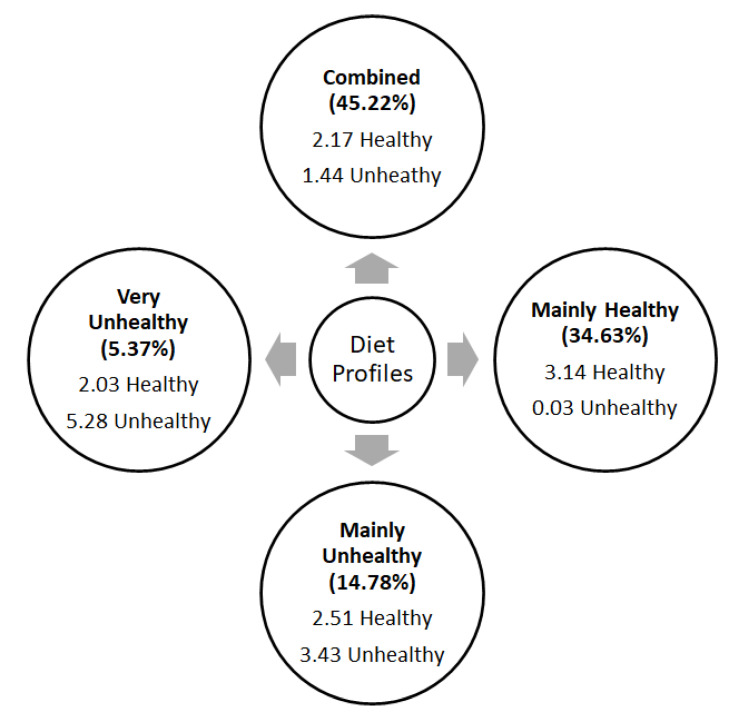
Graphical representation of the mean frequency of healthy and unhealthy daily food consumption in the distinct diet profiles.

Four diet profiles were identified (see Figure 2). The most prevalent diet profile (*n* = 303; 45.22%) corresponds to children who included both healthy (F/V; an average of 2.17 per day, SD = 1.57) and unhealthy (fast food, sugar-sweetened beverages, and candies; an average of 1.44 per day, SD = 0.50) types of food in their diets (class 1). However, it must be highlighted that healthy food consumption in this group is significantly below the average (*p* < 0.001), even though the size of the difference is small (*d* = 0.28). This profile (class 1) was named Combined Diet (47.5% girls). In the remaining three profiles, there is a clear predominance of one type of food over the other. Specifically, two opposing profiles were identified. On the one hand, there are children whose healthy food consumption (an average of 3.14 per day, SD = 1.73) is above average (*n* = 232; 34.63%) and whose unhealthy food consumption (0.03 per day, SD = 0.18) is below average (*p* < 0.001; *d* = 1.42). This profile (class 3) was named Mainly Healthy Diet (50.4% girls). On the other hand, there are children whose diets are characterized by an average consumption of healthy foods (an average of 2.51 per day, SD = 1.78) and a high consumption of unhealthy food (an average of 3.43 per day, SD = 0.50) (*n* = 99; 14.78%). This profile (class 4) was named Mainly Unhealthy Diet (40.4% girls). Lastly, a small group of children was identified to show poor food consumption regarding F/V and high consumption regarding unhealthy foods (*n* = 36; 5.37%). In this group, the consumption of healthy food (an average of 2.03 per day, SD = 1.77) is significantly below average (*p* < 0.1), and consumption of unhealthy food (an average of 5.28 per day, SD = 0.45) is significantly above average (differences with a very large effect size). This profile (class 2) was named Very Unhealthy Diet (36.1% girls). As previously mentioned, the precision in the allocation of children to these groups is excellent, particularly in the profiles where one type of food consumption prevails over the other.

### 3.3. Diet Profiles and Family-Related Outcomes

To address the second goal of the present study, the associations between diet profiles and different family-related variables (i.e., income, food availability, and parental encouragement of healthy eating) were calculated. Results are displayed in Table 4 (descriptive statistics) and Table 5 (relationship between diet profiles and outcome variables).

Overall, data show significant differences between profiles in two of the variables analyzed, i.e., family income and unhealthy food available at home (see the overall test in Table 5). Regarding the other two variables (i.e., parental encouragement and healthy food availability at home), no differences were found. When analyzing the differences between profiles, we found that the largest differences observed were between the Mainly Healthy Diet profile and the profiles with a predominant unhealthy component (i.e., Very Unhealthy Diet and Mainly Unhealthy Diet profiles). Children with a Mainly Healthy Diet profile, when compared to counterparts with unhealthy food consumption profiles (i.e., Mainly Unhealthy Diet and Very Unhealthy Diet), were more likely to be from high-income families and to have less unhealthy food at home.

## 4. Discussion

The present study explored children’s diet profiles according to their reported food consumption and, subsequently, the relationship between the profiles and family-related factors (i.e., family income, food availability at home, and parental encouragement of healthy eating).

Contrary to our initial hypothesis, four consistent and significant diet profiles emerged: Combined Diet, Mainly Healthy Diet, Mainly Unhealthy Diet, and Very Unhealthy Diet. Interestingly, current data indicated that nearly half of the children (45.22%) consumed a Combined Diet, i.e., eating both healthy and unhealthy foods. Another related fact is that most of the participants (94.6%) already included some healthy food (i.e., F/V) consumption in their diets (all profiles except for the Very Unhealthy Diet). Nevertheless, the amount of F/V consumption is below the “five pieces per day” recommendation, even for the children in the Mainly Healthy Diet profile [3]. In fact, even children who follow a Mainly Healthy Diet reported consuming an average of 3.14 F/V per day. Additionally, the general consumption of unhealthy foods (e.g., fast food) was an average of 1.46 pieces per day. Although this is not an ideal amount of unhealthy food consumption, these figures are low compared to results of an Australian sample of 947 elementary-school students [27]. These results reinforce the idea that Portuguese children are not following the dietary recommendations in terms of intake of F/V and unhealthy foods [4,7]. Moreover, the Very Unhealthy Eating profile was mostly comprised of boys. This result is in accordance with the literature that has indicated that boys eat fewer F/V than girls [23,43,44]. A review by Keller et al. [45] presented similar results, and highlighted that the differences in food consumption between boys and girls are likely to be explained by parenting, peer and societal factors.

Present data confirm our initial hypotheses that the Mainly Healthy Diet profile, when compared to the other profiles, was positively and statistically associated with a higher family income, especially when compared to the two unhealthier diet profiles. These findings are in line with previous studies, showing that healthy lifestyle clusters were more likely to be composed of children from high-income families than of children from low-income families [46]. Furthermore, children from low-income families, when compared to children from high-income families, are more prone to report less healthy dietary behaviors [11,12]. Current results also indicated that children in the *Mainly Healthy Diet* profile, when compared with children in the *Very Unhealthy Diet* and *Mainly Unhealthy Diet* profiles, tend to have less access to unhealthy foods at home. Interestingly, data indicated that having more access to healthy foods at home does not seem to be associated with eating more F/V. In fact, children from all diet profiles seem to have on average more F/V at home than unhealthy foods, but this condition by itself does not seem to guarantee a healthy dietary intake.

These findings suggest that the key to promoting healthy eating seems to be more related to the reduction of foods high in fats and sugars available at home rather than increasing the availability of F/V. This result is consistent with the prior literature showing that unhealthy food availability in schools is associated with the purchase of sweets and sugar-sweetened beverages, even when healthy choices are available [47].

Lastly, no relationship was found between children’s diet profiles and parental encouragement of healthy eating. This finding is consistent with data from Pasquale and Rivolta [8] indicating that parental encouragement is relevant to children’s healthy eating, but should be combined with other variables, such as the structure of family meals (e.g., family morals about type and quantity of food available) and parental direct modeling (e.g., eating healthy in front of children: vegetables, soup, or fruit). Grounded on these findings, and on the wisdom of the sarcastic proverb ‘do as I say not as I do’, we believe that the effects of parental verbal encouragement on their children’s diets may be mediated by the parents’ actual food choices.

### 4.1. Implications for Research and Practice

Overall, the present findings may provide contributions for health professionals and educator practices. Individual children’s diet profiles are influenced by sex, income, and food availability at home. These profiles can be used to cluster children into fitting sub-groups, and to tailor and improve healthy eating promotion interventions. For example, children with a Combined Diet could benefit from interventions focused on decreasing their unhealthy food consumption, while children with a Mainly Unhealthy Diet and with a Very Unhealthy Diet could benefit from messages encouraging healthy eating habits. The present study strengthens the relevance of considering the family and their resources in the design of healthy eating interventions for children. A message to reduce unhealthy food availability at home should be a common theme relevant to the three priority profiles (i.e., combined and unhealthy ones).

However, despite the importance of messages instigating good healthy eating habits, that strategy is not enough on its own to promote healthy eating diets in children. Educational interventions should launch strategies to help families with financial difficulties adopt accessible healthy diets. For example, it seems pertinent to increase the price of sugary foods and soft drinks to reduce consumption, but also to combine these actions with social subsidies (e.g., vouchers for families) to increase the consumption of healthy foods [48,49].

Current findings may help understand why data from previous interventions showed limited or small- to medium-sized effects and why the participants’ healthy food consumption remained below the recommendations [10,50,51]. The small effect sizes may not necessarily reflect a lack of or poor strategies promoting healthy eating habits. On the contrary, results may reflect the differential impact of interventions on healthy eating, due to the misalignment between the messages conveyed and the individual child’s diet profile. This suggestion is consistent with findings from educational research. For example, Azevedo et al. [52] found that the impact of a school-based intervention to train self-regulated learning competences was distinct according to participants’ features (e.g., grade retention, engagement in the intervention).

### 4.2. Limitations and Future Directions

Despite the promising results, this study has some limitations. Regarding the measurement of the variables, self-reported measures are likely to lead to recall and social desirability bias, especially in children [53]. To minimize this concern, self-reported measures previously validated in studies with similar samples were used (e.g., [31]). Future research could consider combining self-reported measures with alternative ones, such as observational measures. Moreover, the use of the variable family income, instead of a composed variable to measure socioeconomic status (e.g., income, parent’s academic level, and occupation), may not have fully captured the nuances of this variable in the different diet profiles. Additionally, causal relationships between the diet profiles and family-related factors cannot be established due to the cross-sectional design of the present study. In the future, researchers may conduct longitudinal studies to infer causal relationships between diet profiles and family-related factors, thus conferring a methodological improvement to the research. Furthermore, future studies should consider the influence of distinct aspects such as motivational-related factors (e.g., self-regulation), structure of family meals, parental modeling, and school context. Lastly, despite not being able to generalize the present findings, data of the present study were collected in five Portuguese public schools from rural and urban contexts. This variety of settings is likely to have ensured a wide range of socioeconomic and cultural backgrounds in our sample.

## Figures and Tables

**Table 1:** Descriptive statistics and Pearson’s correlation matrix.

	1	2	3	4	5	6	7
1. Gender	−						
2. Healthy Food	0.12 ^2^	−					
3. Unhealthy Food	−0.08 ^1^	0.13 ^3^	−				
4. Family Income	−0.01	0.03	−0.12 ^2^	−			
5. Parental Encouragement	0.08 ^1^	0.10 ^1^	0.05	0.11 ^2^	−		
6. Food Availability (healthy)	0.06	−0.02	0.03	0.17 ^3^	0.31 ^3^	−	
7. Food Availability (unhealthy)	−0.01	−0.15 ^3^	0.26 ^3^	−0.06	−0.02	0.17 ^3^	−
M	0.47	0	0	2.92	0.80	1.94	1.28
SD	0.50	1	1	1.24	0.27	0.25	0.59
SK	0.13	0.22	1.04	−0.50	−1.49	−3.56	−0.21
KU	−1.99	−0.85	0.46	−1.45	1.74	10.73	−0.58

Gender (0 = boys, 1 = girls). ^1^ *p* < 0.05; ^2^ *p* < 0.01; ^3^ *p* < 0.001. M = mean; SD = standard deviation; SK = skewness; KU = kurtosis.

**Table 2:** Statistics of two, three, and four latent model fitting.

	Two Classes	Three Classes	Four Classes
AIC	3654.288	3582.645	3554.535
BIC	3685.839	3628.718	3626.651
SSA-BIC	3663.614	3595.967	3575.850
Entropy	0.861	0.880	0.858
LMRT-Test (*p*)	152.646 (<0.0001)	73.859 (<0.0001)	35.390 (0.0010)
N < 5%	0	0	0

AIC = Akaike Information Criterion; BIC = Schwarz Bayesian Information Criterion; SSA-BIC = BIC adjusted for the sample size; LMRT-Test = adjusted Lo–Mendell–Rubin maximum likelihood ratio test.

**Table 3:** Descriptive statistics of the four diet profiles.

	Estimate	S.E.	T	*p*	LO5%	HI5%
Class 1: Combined diet						
Healthy food	−0.228	0.064	−3.579	<0.001	−0.333	−0.123
Unhealthy food	0.043	0.054	0.800	0.423	−0.046	0.132
Class 2: Very unhealthy diet						
Healthy food	−0.301	0.170	−1.770	0.077	−0.581	−0.021
Unhealthy food	2.534	0.060	42.391	<0.001	2.436	2.632
Class 3: Mainly healthy diet						
Healthy food	0.260	0.076	3.440	.001	0.136	0.385
Unhealthy food	−0.843	0.056	−14.971	<0.001	−0.936	−0.751
Class 4: Mainly unhealthy diet						
Healthy food	−0.016	0.107	−0.147	0.883	−0.191	0.160
Unhealthy food	1.319	0.039	34.023	<0.001	1.255	1.383

LO5% and HI5% are the confidence intervals. Combined diet (*n* = 303; 45.22%), Very unhealthy diet (*n* = 36; 5.37%), Mainly healthy diet (*n* = 232; 34.62%), Mainly unhealthy diet (*n* = 99; 14.77%).

**Table 4:** Means and standard errors.

	Combined Diet	Mainly Healthy Diet	Mainly Unhealthy Diet	Very Unhealthy Diet
Family Income				
Mean	2.925	3.032	2.733	2.453
S.E.	0.082	0.076	0.132	0.211
Parental Encouragement				
Mean	0.814	0.784	0.794	0.847
S.E.	0.017	0.018	0.026	0.039
Food Availability (healthy)				
Mean	1.939	1.930	1.930	1.971
S.E.	0.016	0.016	0.026	0.028
Food Availability (unhealthy)				
Mean	1.313	1.139	1.466	1.663
S.E.	0.041	0.036	0.061	0.081

Family income (1 = extremely low income; 2 = very low income; 3 = low income; 4 = medium to high income); Parental encouragement of healthy eating (0 = no encouragement, 1 = high encouragement); Food availability (healthy) (0 = absence of healthy food, 1 = presence of fruit or vegetables, 2 = presence of both fruit and vegetables); Food availability (unhealthy) (0 = absence of unhealthy food, 1 = presence of sugars or fats, 2 = presence of both sugars and fats).

**Table 5:** Relationship between diet profiles and outcome variables.

	Classes	χ^2^	*p*	Cohen’ *d*	Classes	χ^2^	*p*	Cohen’ *d*
Family Income	Overall test	9.558	0.023	0.241				
	1 vs. 2	4.353	0.037	0.228	2 vs. 3	6.669	0.010	0.319
	1 vs. 3	0.825	0.364	-	2 vs. 4	1.248	0.264	-
	1 vs. 4	1.506	0.220	-	3 vs. 4	3.851	0.050	0.217
Parental Encouragement	Overall test	2.388	0.496	-				
	1 vs. 2	0.566	0.452	-	2 vs. 3	2.096	0.148	-
	1 vs. 3	1.423	0.233	-	2 vs. 4	1.246	0.264	-
	1 vs. 4	0.445	0.505	-	3 vs. 4	0.099	0.753	-
Food Availability (healthy)	Overall test	1.719	0.633	-				
	1 vs. 2	1.010	0.315	-	2 vs. 3	1.649	0.199	-
	1 vs. 3	0.153	0.696	-	2 vs. 4	1.156	0.282	-
	1 vs. 4	0.094	0.759	-	3 vs. 4	0.000	0.996	-
Food Availability (unhealthy)	Overall test	51.486	<0.001	0.577				
	1 vs. 2	14.931	<0.001	0.302	2 vs. 3	35.435	<0.001	0.473
	1 vs. 3	9.134	0.003	0.235	2 vs. 4	3.716	0.050	0.149
	1 vs. 4	4.346	0.037	0.162	3 vs. 4	21.293	<0.001	0.362

Class 1 (combined diet: *n* = 303, 45.22%), Class 2 (very unhealthy diet: *n* = 36, 5.37%), Class 3 (mainly healthy diet: *n* = 232, 34.62%), Class 4 (mainly unhealthy diet: *n* = 99, 14.77%).

## Data Availability

Research data are not shared.
